# Spatiotemporal inequality and financial toxicity of leukemia in post-poverty China: a national analysis of 832 counties (2019–2024)

**DOI:** 10.3389/fpubh.2025.1611102

**Published:** 2025-09-26

**Authors:** Wenping Li, Zhiyu Lv, Mengdi Chen, Dong Xia, Jiayue Wang, Jiapeng Chen, Lulu Zhang

**Affiliations:** ^1^Department of Health Services, Naval Medical University, Shanghai, China; ^2^China Population and Development Research Center, Beijing, China

**Keywords:** leukemia, China, poverty-alleviated regions, spatiotemporal analysis, financial toxicity

## Abstract

**Background:**

Leukemia remains a critical public health challenge in China’s post-poverty regions, where high treatment costs perpetuate the “disease-poverty” trap. Despite nationwide efforts to improve healthcare access, the evolving spatiotemporal dynamics and economic burden of leukemia in these regions remain understudied.

**Methods:**

Using population-based data from China’s Health Poverty Alleviation Platform (2019–2024), we analyzed 97,472 leukemia cases across 832 poverty-alleviated counties. Age/sex-standardized incidence and mortality rates were calculated using 2020 census data. Spatiotemporal trends were evaluated via Joinpoint regression, and spatial clustering was mapped through global/local Moran’s I and Getis-Ord Gi* analyses. The economic burden was assessed by Out-Of-Pocket (OOP) payment ratios and costs.

**Results:**

Longitudinal analysis of 97,472 leukemia cases across 832 Chinese poverty-alleviated counties (2019–2024) revealed: (1) Significant reductions in age-standardized incidence (AAPC = −59.4%, *p* = 0.015) and mortality (AAPC = −67.5%, *p* = 0.012), with persistently higher male incidence (χ^2^ = 1554.4, *p* < 0.001); (2) Spatiotemporal transition from Northeast/Central clustering (Moran’s I > 0.38, *p* < 0.001; 2019–2021) to Western hotspot expansion (Getis-Ord Gi*, *p* < 0.001; 2022–2024), indicative of improved diagnostic coverage; (3) Severe financial toxicity in Eastern China (median OOP ratio = 39.7%, approaching WHO catastrophic thresholds) and high absolute OOP cost clustering in Central regions, driven by therapy costs and insurance fragmentation.

**Conclusion:**

While China’s poverty alleviation policies effectively reduced the leukemia burden, persistent regional disparities and financial toxicity demand targeted interventions. The westward hotspot migration post-2022 marks a diagnostic catch-up in resource-limited regions. Crucially, elevated male/youth incidence necessitates targeted screening in emerging clusters, while diverging financial toxicity demands region-specific solutions: for Eastern China’s catastrophic OOP ratios (39.7%), reform must prioritize novel-therapy reimbursement; Central China’s cost-clustering urges cross-provincial care networks to offset abandonment risks.

## 1 Introduction

Leukemia, a group of chronic malignant neoplastic diseases characterized by the uncontrolled proliferation of abnormal white blood cells, exerts profound and multifaceted impacts on patients-disrupting physical health, triggering psychological distress, and imposing heavy economic burdens (1, 2). As a major global public health challenge (3), its burden exhibits striking geographic heterogeneity, particularly in low- and middle-income settings where healthcare access remains constrained (4). In China, while the overall leukemia incidence is categorized as intermediate compared to global benchmarks (5), the disease’s distribution and impact in poverty-alleviated regions have long been underappreciated. This oversight is critical, as impoverished populations in these areas face a unique conundrum: limited access to primary care amplifies leukemia risk (4), and the disease itself acts as a potent driver of both persistent poverty and poverty recurrence-a “disease-poverty trap” that undermines the sustainability of China’s anti-poverty achievements.

China’s “Precision Poverty Reduction” strategy, launched in the late 2010s, marked a pivotal step in addressing health-related poverty. By reducing Out-Of-Pocket (OOP) medical expenses and curbing catastrophic healthcare costs, the policy significantly improved healthcare accessibility in impoverished counties (6). However, since China officially declared the elimination of absolute poverty in 2020, emerging evidence suggests that health-related financial burdens persist. Many families in poverty-alleviated regions still face the threat of returning to poverty due to high leukemia treatment costs. This highlights a critical gap between policy implementation and sustained health equity. Compounding this challenge, the epidemiological characteristics of leukemia in these newly poverty-free areas-including trends in incidence, mortality, and their spatiotemporal dynamics-have not been systematically quantified. Prior studies on Chinese leukemia burden have either focused on national or provincial scales (2, 5) or pre-poverty alleviation periods (4), leaving a void in understanding how the disease’s burden has evolved after the lifting of absolute poverty. Similarly, research on leukemia’s economic impact in these regions has been largely unidimensional, often focusing solely on OOP costs without integrating relative financial toxicity metrics (e.g., OOP ratios relative to household health expenditure), a limitation that obscures the true extent of economic vulnerability.

To fill these gaps, we conducted a multidimensional analysis of leukemia in 832 poverty-alleviated counties across China’s four major economic regions (Northeast, Central, East, and West) from 2019 to 2024. Leveraging population-based data from China’s Health Poverty Alleviation Big Data Platform-one of the largest datasets on post-poverty health outcomes to date-we analyzed 97,472 newly diagnosed leukemia cases. Our study aimed to address three core research questions: (1) How have leukemia incidence and mortality rates changed longitudinally in post-poverty counties, and do these trends differ by age, sex, or region? (2) What are the spatiotemporal dynamics of leukemia burden-including spatial clustering and regional shifts-across the study period? (3) How is the economic burden of leukemia (measured by both absolute OOP costs and relative OOP ratios) distributed across regions, and what factors drive these disparities?

## 2 Materials and methods

### 2.1 Data sources

Incidence and mortality data (2019–2024) were obtained from China’s Health Poverty Alleviation Big Data Platform, covering 832 counties officially removed from poverty registries(application required). Population denominators were adjusted using the 2020 Chinese National Census. County-level boundaries were sourced from the National Geomatics Center (Approval No. GS [2024]0650). The division of China’s four major economic regions (Northeast, Central, East, and West) was based on the Guiding Opinions of the Communist Party of China Central Committee and the State Council on Promoting Coordinated Regional Development.

### 2.2 Statistical methods

Time trends in leukemia incidence and mortality were analyzed using the Joinpoint regression model (Joinpoint Regression Program, Version 4.9.1.0, National Cancer Institute). Joinpoint regression is specifically designed to identify significant temporal turning points (joinpoints). These joinpoints refer to calendar years when the slope of a trend (e.g., incidence or mortality rate) exhibits a statistically significant change. The detection of such joinpoints indicates shifts in underlying disease dynamics, which may correlate with contextual factors including public health interventions or environmental exposures. To prevent model overfitting, we restricted the maximum number of turning points to two based on the sample size and study period of 6 years. The optimal number of turning points was determined through a permutation test (Permutation Test, 4,500 replications) at a significance level of *α* = 0.05. The Annual Percentage Change (APC) and the Average Annual Percentage Change (AAPC) were calculated for each period, with confidence intervals estimated through 10,000 Monte Carlo simulations. We compared different models with a maximum of three turning points (ranging from 0 to 3) and ultimately selected the model with the smallest Bayesian Information Criterion (BIC) value as the optimal solution.

Global spatial autocorrelation analysis was performed to examine the spatial distribution of three indicators: incidence rate, average out-of-pocket cost, and OOP ratio. This analysis used the ArcGIS 10.8 Spatial Statistics Toolkit, with a fixed bandwidth of 200 km and the Queen neighborhood criterion. We generated spatial distribution maps and optimized hotspot analysis maps. The significance of Moran’s I was evaluated via a 999-time Monte Carlo permutation test (*α* = 0.05).

Statistical methods employed included the chi-square test to assess differences in group incidence rates, with two-by-two comparisons adjusted using the Bonferroni method (*α* = 0.05). In accordance with the American Statistical Association guidelines, homogeneous subgroups were differentiated using superscript letters (a, b, c, d), where groups sharing common letters indicate no statistically significant difference (*p* ≥ 0.05).

### 2.3 Standardization

To ensure the comparability of leukemia incidence and mortality rates across different years, regions, age groups, and sexes-while accounting for variations in population structure (e.g., age/sex distribution differences among the 832 poverty-alleviated counties)-we applied direct standardization using the 2020 Chinese National Census data as the reference population. This approach eliminates the confounding effect of population composition, allowing for accurate temporal and spatial trend analyses of leukemia burden. Below are the key standardized metrics used in this study, along with their definitions and calculation formulas:

- Age-Standardized Incidence Rate (ASIR): A rate adjusted to a standard age distribution to reflect the incidence of leukemia that would be observed if different populations had the same age structure. It is used to compare leukemia incidence across groups with varying age compositions.

$$\text{ASIR}=(\frac{\sum \mathrm{Age}-\text{stratum}\;\text{incident cases}}{\text{Total}\;\text{population}})\times \lambda_{\left[\mathrm{age}\right]}\times 100,000$$

- Gender-Standardized Incidence Rate (GSIR): A rate adjusted to a standard sex distribution to compare leukemia incidence across populations with different male-to-female ratios.

$$\text{GSIR}=(\frac{\sum \mathrm{Sex}-\text{stratum}\;\text{incident cases}}{\text{Total}\;\text{population}})\times \lambda_{\left[\mathrm{sex}\right]}\times 100,000$$

- Age-Standardized Mortality Rate (ASMR): A mortality rate adjusted to a standard age distribution, enabling unbiased comparisons of leukemia mortality across groups with differing age structures.

$$\text{ASMR}=(\frac{\sum \mathrm{Age}-\text{stratum Deaths}}{\text{Total}\;\text{population}})\times \lambda_{\left[\mathrm{age}\right]}\times 100,000$$

- Gender-Standardized Mortality Rate (GSMR): A mortality rate adjusted to a standard sex distribution, used to compare leukemia mortality across populations with varying sex compositions.

$$\text{GSMR}=(\frac{\sum \mathrm{Sex}-\text{stratum}\;\text{Deat}\mathrm{hs}}{\text{Total}\;\text{population}})\times \lambda_{\left[\mathrm{sex}\right]}\times 100,000$$

λ: Standardized proportion according to the results of the 2020 Chinese National Census.

- Out-of-Pocket (OOP) Ratio: A relative indicator of financial burden, reflecting the proportion of a household’s total health expenditure allocated to leukemia-specific direct medical costs. It helps assess the relative economic pressure of leukemia treatment on households, with values approaching or exceeding the World Health Organization (WHO) defined catastrophic threshold (40%), indicating severe financial toxicity.

$$\mathrm{OOP}\;\text{ratio}=(\;\frac{\text{Annual}\;\text{leukemia related}\;\mathrm{OOP}\;\text{cost}}{\text{Total}\;\text{household health expenditure}})\times 100\%$$

- Out-of-Pocket (OOP) cost: Sum of direct medical expenditures (including diagnostics, medication, hospitalization) within leukemia-specific episodes, excluding non-health consumption. Only direct medical costs were captured; indirect costs (e.g., income loss, caregiving, transportation) were excluded due to data constraints.”

## 3 Results

### 3.1 Descriptive statistics

Between 2019 and 2024, the total number of new cases was 97,472, with a cumulative crude incidence rate of 896.52 per 100,000 individuals, demonstrating a significant yearly decreasing trend in the annual crude incidence rate (average annual percentage of decline AAPC = −59.79, *p* = 0.014; Table 1). Based on data from the Seventh Population Census of China in 2020, the age-standardized results indicated that the incidence rate declined annually from 2019 (132.78 per 100,000; AAPC = −59.44, *p* = 0.015) to 2024 (3.38 per 100,000), resulting in a cumulative incidence rate of 415.59 per 100,000 (Table 1). Gender-standardized analyses also revealed a yearly decrease in incidence from 143.84 per 100,000 in 2019 (AAPC = −59.54, *p* = 0.017) to 4.18 per 100,000 in 2024, with a cumulative incidence of 451.70 per 100,000 (Table 1). Between 2019 and 2024, a cumulative total of 117,243 deaths were recorded, yielding a cumulative crude mortality rate of 1,031.28 per 100,000, which exhibited a significant downward trend in the annual crude mortality rate (AAPC = −64.73, *p* = 0.026; Table 1). According to the 2020 Chinese census data, the age-standardized results showed that the mortality rate declined each year from 2019 (125.62 per 100,000; AAPC = −67.53, *p* = 0.012) to 2024 (1.44 per 100,000; Table 1). Meanwhile, gender-standardized analysis results also indicated a yearly decrease in the mortality rate (AAPC = −66.89, *p* = 0.013) from 2019 (135.93 per 100,000) to 1.74 per 100,000 in 2024 (Table 1).

**Table 1:** Annual and cumulative leukemia incidence and mortality rates (per 100,000 population) in 832 poverty-alleviated counties of China (2019–2024).

Year	Incidence				Mortality			
	Account	Crude rate	ASIR	GSIR	Account	Crude rate	ASMR	GSMR
2019	23,966	285.87	132.78	143.84	22,747	271.33	125.62	135.93
2020	35,176	324.38	150.88	163.19	34,304	316.34	147.14	159.07
2021	34,040	257.34	118.63	129.31	33,505	253.29	116.84	127.38
2022	1,393	9.97	4.45	4.97	25,675	183.68	2.74	3.00
2023	1893	12.34	5.48	6.21	596	3.89	1.76	1.99
2024	1,003	6.62	3.38	4.18	416	2.75	1.44	1.74
Total	97,472	896.52	415.59	451.70	117,243	1031.28	395.54	429.11
AAPC	-	−59.79	−59.44	−59.54	-	−64.73	−67.53	−66.89
95%CI	-	−78.06	−78.15	−78.00	-	−84.71	−84.26	−84.00
		−26.29	−24.72	−25.58		−18.62	−33.05	−31.48
*p*	-	0.014	0.015	0.017	-	0.026	0.012	0.013

All standardized rates were adjusted using the 2020 Chinese National Census data as the reference population to eliminate confounding by population age/sex structure. ASIR, Age-Standardized Incidence Rate; GSIR, Gender-Standardized Incidence Rate; ASMR, Age-Standardized Mortality Rate; GSMR, Gender-Standardized Mortality Rate; AAPC, Average Annual Percentage Change; CI, Confidence Interval. AAPC and 95% CI were calculated via Joinpoint Regression (Version 4.9.1.0, National Cancer Institute); *p* < 0.05 indicates a statistically significant annual trend in incidence/mortality.

**Figure 1:**
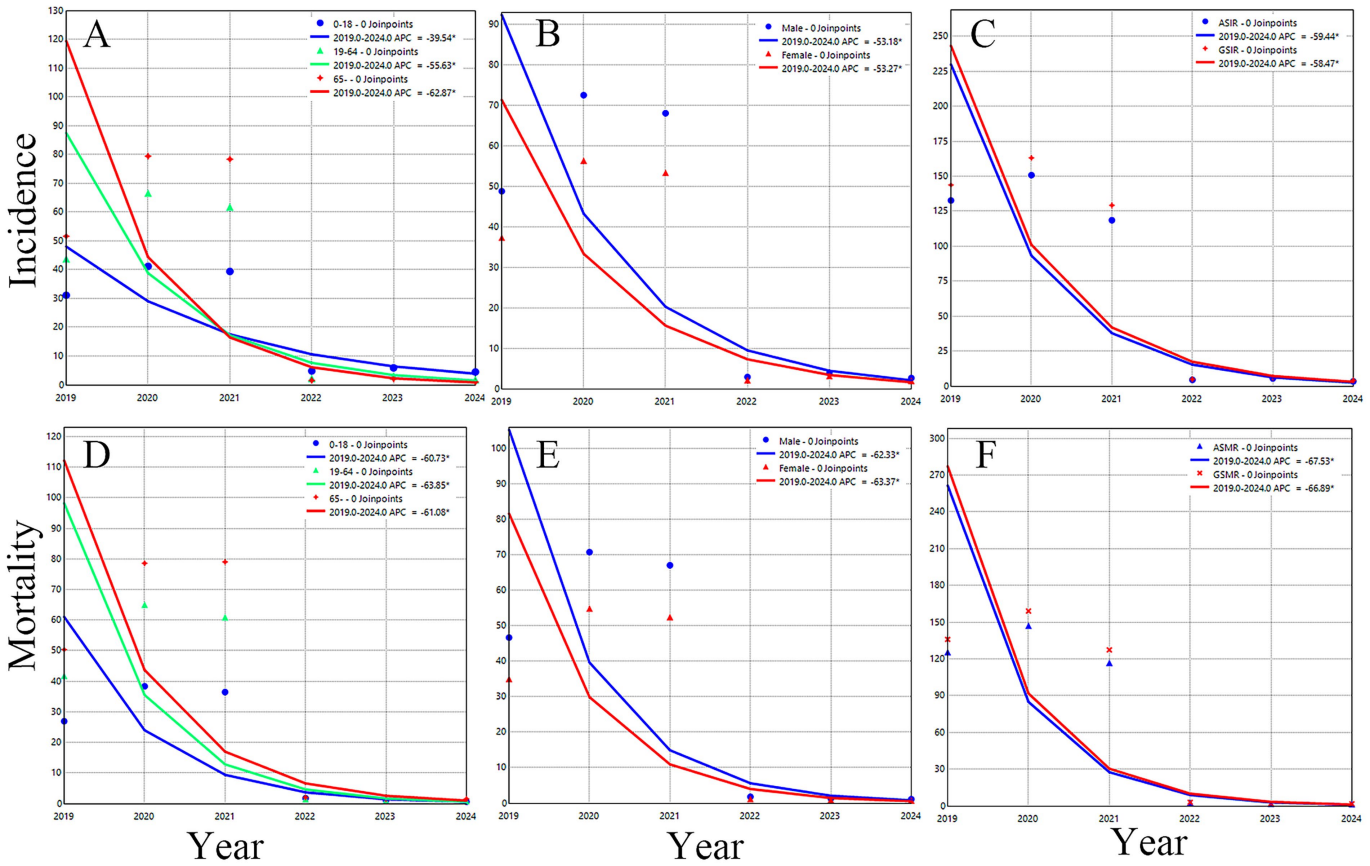
Joinpoint regression analysis of leukemia incidence and mortality rates in 832 poverty-alleviated counties (2019–2024). Incidence rates for age-stratified groups **(A)**, sex-stratified groups **(B)**, and standardized groups **(C)** exhibited statistically significant downward trends; Mortality rates for age-stratified groups **(D)**, sex-stratified groups **(E)**, and standardized groups **(F)** also demonstrated statistically significant downward trajectories. ASIR, Age-Standardized Incidence Rate; ASMR, Age-Standardized Mortality Rate; APC, Annual Percentage Change.

The results of the JoinPoint incidence rate calculations indicated a decreasing trend in the incidence rates across various age and sex groups, as well as the overall standardized incidence rate (Figure 1). Both the ASIR and GSIR exhibited a significant downward trend, with APC values of −59.44 and −58.47, respectively (Figure 1C). Between 2019 and 2024, a total of 11,777 cases were reported in the juvenile group, 61,649 in the adult group, and 24,282 in the older adults group, with statistically significant differences in incidence rates among age groups in each year(χ^2^ > 200, *p* < 0.001; Table 2). In 2019–2021, the incidence rates were highest in older population, followed by the adult and juvenile groups; and in 2022–2024, the incidence rates were highest in the juvenile, followed by the adult and age group of people over 65 (Table 2). All age groups incidence rates showed a significant decline, with APC values of −39.54,-55.63 and −62.87, respectively (Figure 1A). A total of 58,467 cases were reported in males and 39,250 cases in females, with a male-to-female sex ratio of 1.49:1, and the incidence rate in males was higher than that in females in each year (χ^2^ > 15, *p* < 0.001), and the difference was statistically significant (Table 3). Both male and female incidence rates showed a significant decline, with APC values of −53.27 and −53.18, respectively (Figure 1B).

**Table 2:** Age-stratified leukemia incidence rates (per 100,000 population) in 832 poverty-alleviated counties of China (2019–2024).

Year	0–18		19–64		65-		χ^2^	*p*
	Cases	Incidence	Cases	Incidence	Cases	Incidence		
2019	2,638	31.24^a^	15,257	43.69^b^	6,071	51.39^c^	462.273	<0.001
2020	3,685	40.93^a^	22,481	66.73^b^	9,010	79.79^c^	1196.261	
2021	3,815	39.74^a^	21,456	62.03^b^	8,769	78.32^c^	1257.02	
2022	503	4.83^a^	776	2.25^b^	151	1.55^c^	255.423	
2023	636	5.93^a^	1,063	3.11^b^	195	2.12^c^	244.261	
2024	500	4.46^a^	617	1.81^b^	86	0.99^c^	336.916	
Total	11,777	127.13^a^	61,649	179.63^b^	24,282	214.17^c^	3820.557	

Incidence rates were standardized using the 2020 Chinese National Census data to account for age-structure differences across counties. The χ^2^ test was used to compare incidence rate differences among age groups; *p* < 0.001 indicates overall significant differences across the three age groups in each year. Superscript letters (^a^, ^b^, ^c^) indicate homogeneous subgroups via the Bonferroni *post-hoc* test: groups with different letters have statistically significant differences (*p* < 0.05), while groups with the same letter have no significant difference (*p* ≥ 0.05).

**Table 3:** Sex-stratified leukemia incidence rates (per 100,000 population) in 832 poverty-alleviated counties of China (2019–2024).

Year	Male		Female		χ^2^	*p*
	Cases	Incidence	Cases	Incidence		
2019	14,438	48.71	9,528	37.31	410.601	<0.001
2020	21,052	72.68	14,124	56.46	542.433	
2021	20,263	68.30	13,777	53.57	486.826	
2022	848	2.91	535	2.10	34.716	
2023	1,100	3.81	793	3.15	16.542	
2024	766	2.65	493	1.96	27.577	
Total	58,467	199.06	39,250	154.55	1554.355	

Incidence rates were gender-standardized using the 2020 Chinese National Census data to eliminate confounding by sex-structure variations across counties. The χ^2^ test was used to compare incidence rate differences between males and females; *p* < 0.001 indicates a statistically significantly higher incidence rate in males than in females in each year. The overall male-to-female incidence ratio was 1.49:1 (total male cases/total female cases = 58,467/39250), consistent with the sex-specific trend observed annually.

The results of the JoinPoint mortality calculations indicate a decreasing trend in the mortality rates across various age and sex groups, as well as a decline in the overall standardized mortality rate. The adult group experienced the most significant decrease, followed by the juvenile and age group of people over 65, with respective declines of −63.85, −61.08, and −60.73 (Figure 1D). Male mortality decreased more than female mortality, with rates of −62.33 and −63.37, respectively (Figure 1E). Both the ASMR and the GSMR exhibited a downward trend, with values of −67.53 and −66.89, respectively (Figure 1F).

### 3.2 Leukemia incidence distribution patterns

**Figure 2:**
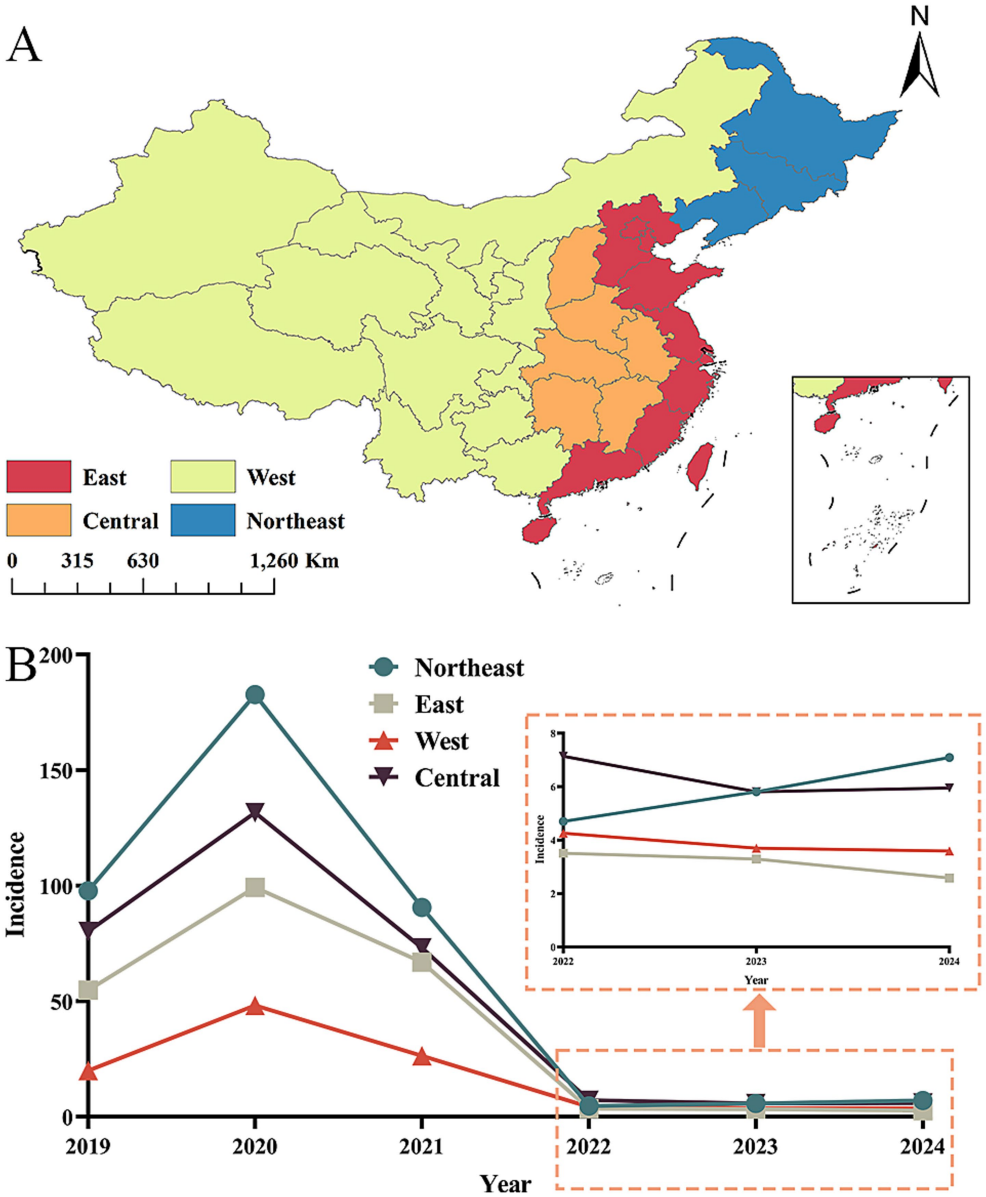
Division of China’s four major economic regions and temporal trends in leukemia incidence (2019–2024). **(A)** Administrative division of the four major economic regions. **(B)** Temporal trends in crude incidence rates (per 100,000) across the four regions, regions showed a notable decline post-2020. 2019–2021: Northeast China had the highest incidence (2020 peak), followed by Central, East, and West China. 0.2022–2024: Incidence in all regions dropped to <10 per 100,000, with the regional hierarchy narrowing. The division of China’s four major economic regions (Northeast, Central, East, and West) was based on the Guiding Opinions of the Communist Party of China Central Committee and the State Council on Promoting Coordinated Regional Development. The 2020 peak in the Northeast/Central regions may reflect backlogged case identification following health poverty-alleviation interventions.

**Figure 3:**
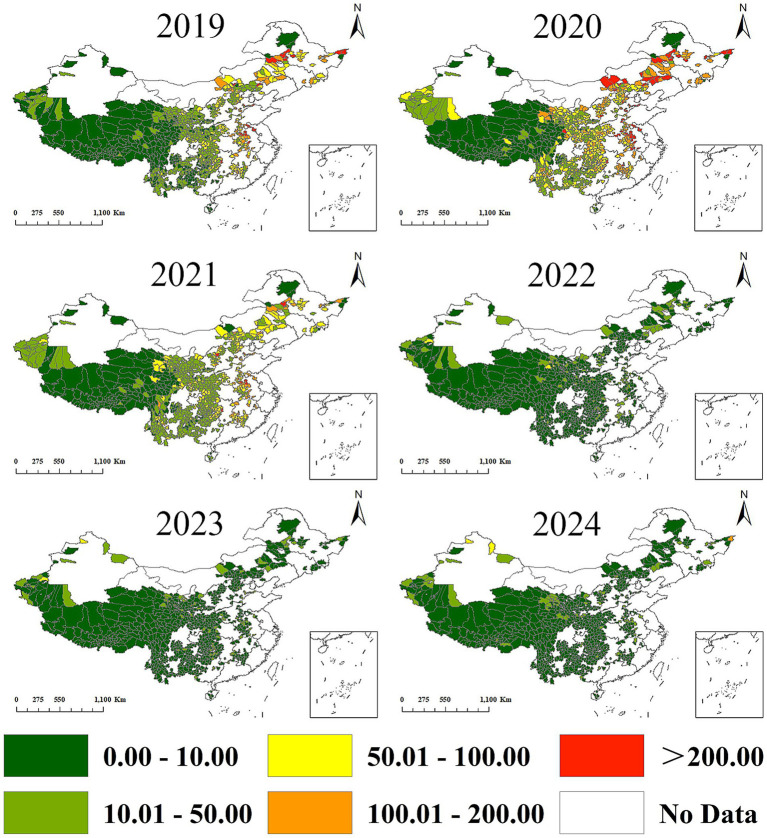
Spatiotemporal distribution of leukemia incidence in 832 poverty-alleviated counties (2019–2024). The spatial pattern of high-incidence counties shifted dynamically over the study period.2019–2021: High-incidence counties were densely clustered in Northeast China and scattered in Central China. East China had sporadic high-incidence counties, while West China had almost no high-incidence areas.2022–2024: The number of high-incidence counties decreased sharply across all regions. Northeast and Central China retained only isolated high-incidence counties, while West China began to show a small number of low-level high-incidence counties -consistent with improved diagnostic coverage in resource-limited regions. County boundaries were sourced from the National Geomatics Center (Approval No. GS [2024]0650).

From 2019 to 2024, leukemia incidence among impoverished populations in China’s four major economic regions displayed significant spatial heterogeneity (χ^2^ = 28,385.27, *p* < 0.001), exhibiting a distinct regional hierarchy: Northeast > Central > East > West (Table 4). The 2019–2020 period revealed differential patterns, with both the Northeast and Eastern regions demonstrating elevated incidence rates. The Northeast particularly showed concentrated high-incidence clusters in 2020, whereas the Central region exhibited mixed medium-to-medium-high incidence counties without notable clustering. Conversely, the Western region consistently maintained low incidence levels throughout. Subsequent analysis (2020–2024) showed all regions transitioning toward predominantly low-incidence distribution patterns (Figures 2, 3).

**Table 4:** Regional leukemia incidence rates (per 100,000 population) across four major economic regions of China (2019–2024, 832 poverty-alleviated counties).

Year	Northeast		Central		East		West		χ^2^	*p*
	Cases	Incidence	Cases	Incidence	Cases	Incidence	Cases	Incidence		
2019	634	97.79^a^	7,236	80.23^b^	1,141	54.74^c^	12,972	20^d^	10841.142	<0.001
2020	1,150	182.70^a^	17,253	131.64^b^	1971	99.26^c^	20,236	48.17^d^	11077.353	
2021	600	90.60^a^	9,492	73.13^b^	1,317	66.93^c^	12,213	26.42^d^	6473.739	
2022	29	4.70^a^	1,553	7.14^b^	67	3.51^c^	1,112	4.26^d^	188.253	
2023	35	5.81^a^	1,341	5.81^b^	62	3.29^c^	889	3.70^d^	118.74	
2024	42	7.09^a^	1,302	5.95^b^	49	2.58^c^	903	3.59^d^	164.563	
Total	2,490	66.27^a^	38,177	51.25^b^	4,607	39.32^c^	48,325	17.60^d^	28385.274	

The division of China’s four major economic regions (Northeast, Central, East, and West) was based on the Guiding Opinions of the Communist Party of China Central Committee and the State Council on Promoting Coordinated Regional Development. The χ^2^ test was used to compare incidence rate differences across regions; *p* < 0.001 indicates overall significant regional disparities in each year. Superscript letters (^a^, ^b^, ^c^, ^d^) indicate homogeneous subgroups via the Bonferroni *post-hoc* test: regions with different letters have statistically significant differences (*p* < 0.05), while regions with the same letter have no significant difference (*p* ≥ 0.05).

**Figure 4:**
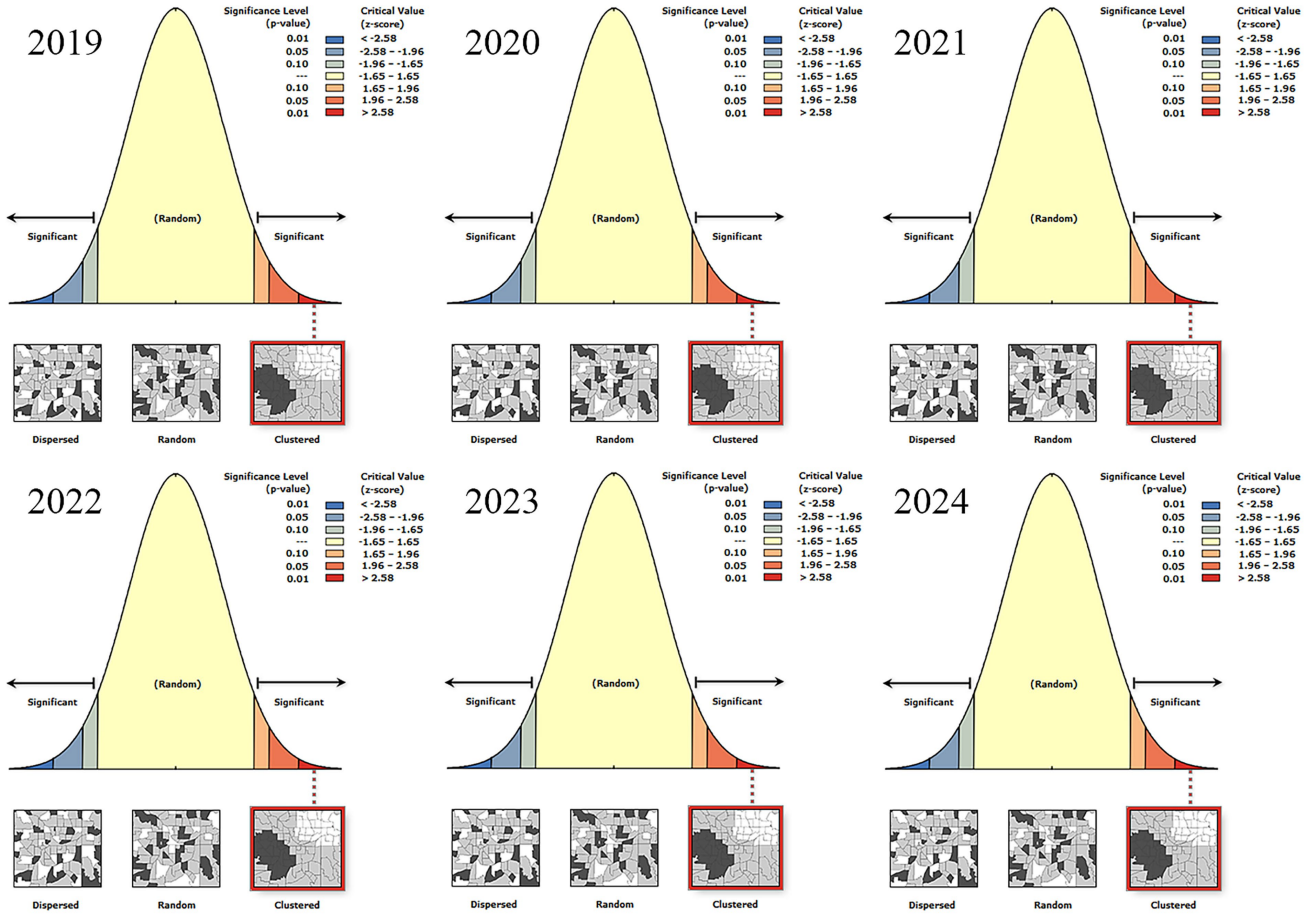
Global Moran’s I statistics for spatial autocorrelation of leukemia incidence (2019–2024). Leukemia incidence exhibited statistically significant spatial clustering in all study years (Monte Carlo permutation test, 999 replications, *p* < 0.01), with a gradual weakening of clustering intensity over time. Positive Moran’s I values indicate spatial aggregation (high-incidence counties adjacent to other high-incidence counties, and low-incidence counties adjacent to low-incidence counties); fixed bandwidth = 200 km, Queen neighborhood criterion.

Significant spatial aggregation patterns persisted across all study years, as evidenced by positive global autocorrelation indices: 2019 (Moran’s I = 0.245, *p* < 0.001), 2020 (Moran’s I = 0.383, *p* < 0.001), 2021 (Moran’s I = 0.386, *p* < 0.001), 2022 (Moran’s I = 0.139, *p* < 0.001), 2023 (Moran’s I = 0.143, *p* < 0.001), and 2024 (Moran’s I = 0.076, *p* < 0.001; Figure 4).

**Figure 5:**
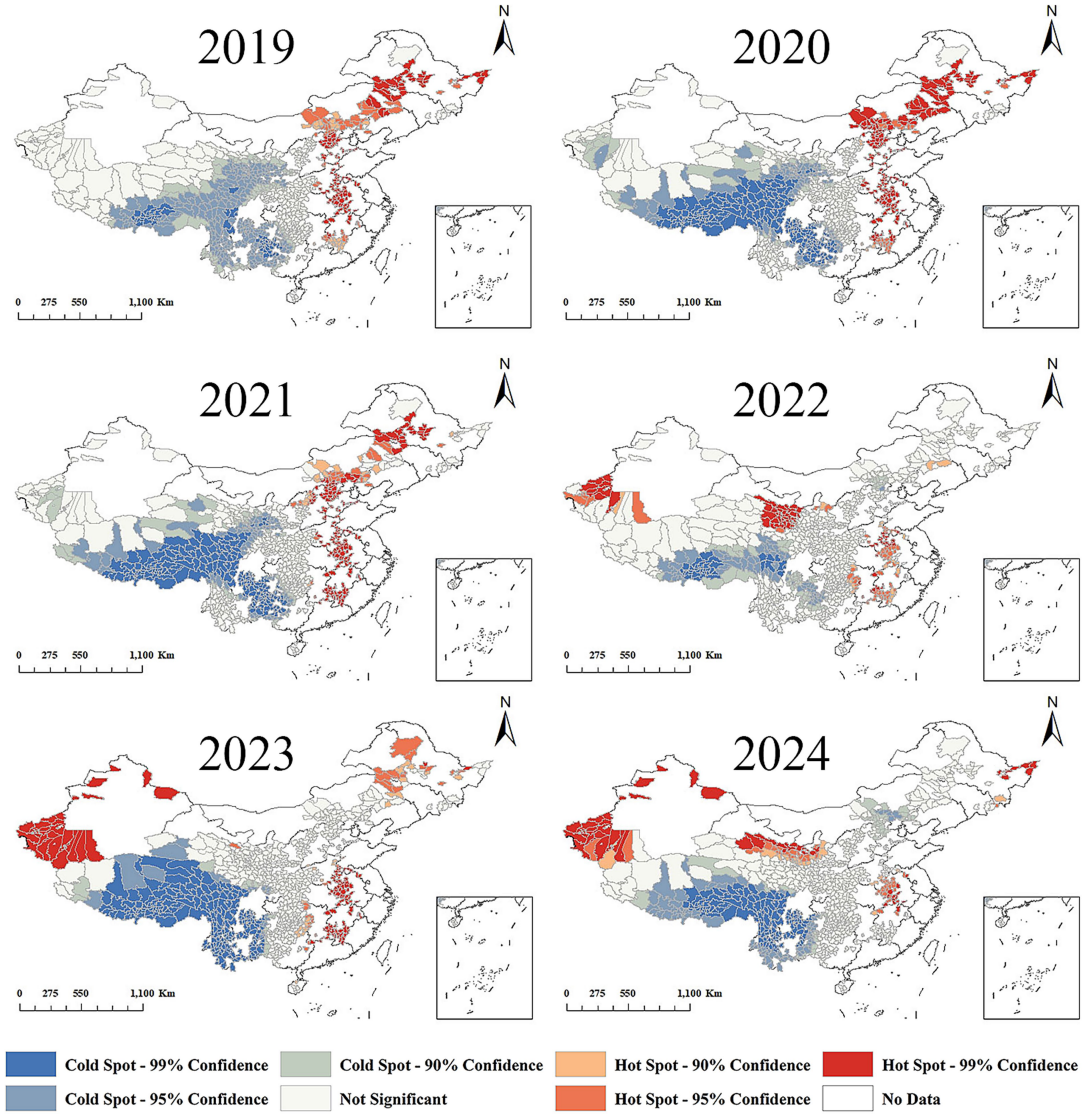
Getis-Ord Gi* statistics for local spatial autocorrelation of leukemia incidence (2019–2024). Shift of leukemia hotspots from Northeast/Central to Western China after 2022. 2019–2021: Hotspots were concentrated in Northeast China and parts of Central China; coldspots dominated in West China with gradual spatial contraction. 2022–2024: Hotspots in Northeast/Central China attenuated significantly; new hotspots emerged and expanded in West China. Coldspots persisted in remote western areas but shrank in size. The post-2022 westward shift in hotspots reflects improved diagnostic capacity in Western China rather than a true increase in leukemia burden, aligning with the rollout of health poverty-alleviation interventions in resource-limited regions.

Getis-Ord Gi* analysis identified distinct spatial–temporal patterns: 2019–2021 witnessed concentrated leukemia hotspots in Northeast and Central China, while Coldspots predominated in Central-West regions with progressive spatial contraction (Figure 5). Subsequent years (2022–2024) showed dynamic spatial evolution - Eastern hotspots demonstrated progressive attenuation, whereas Western regions developed novel hotspot clusters showing significant areal expansion (Figure 5).

### 3.3 Economic burden distribution patterns

**Figure 6:**
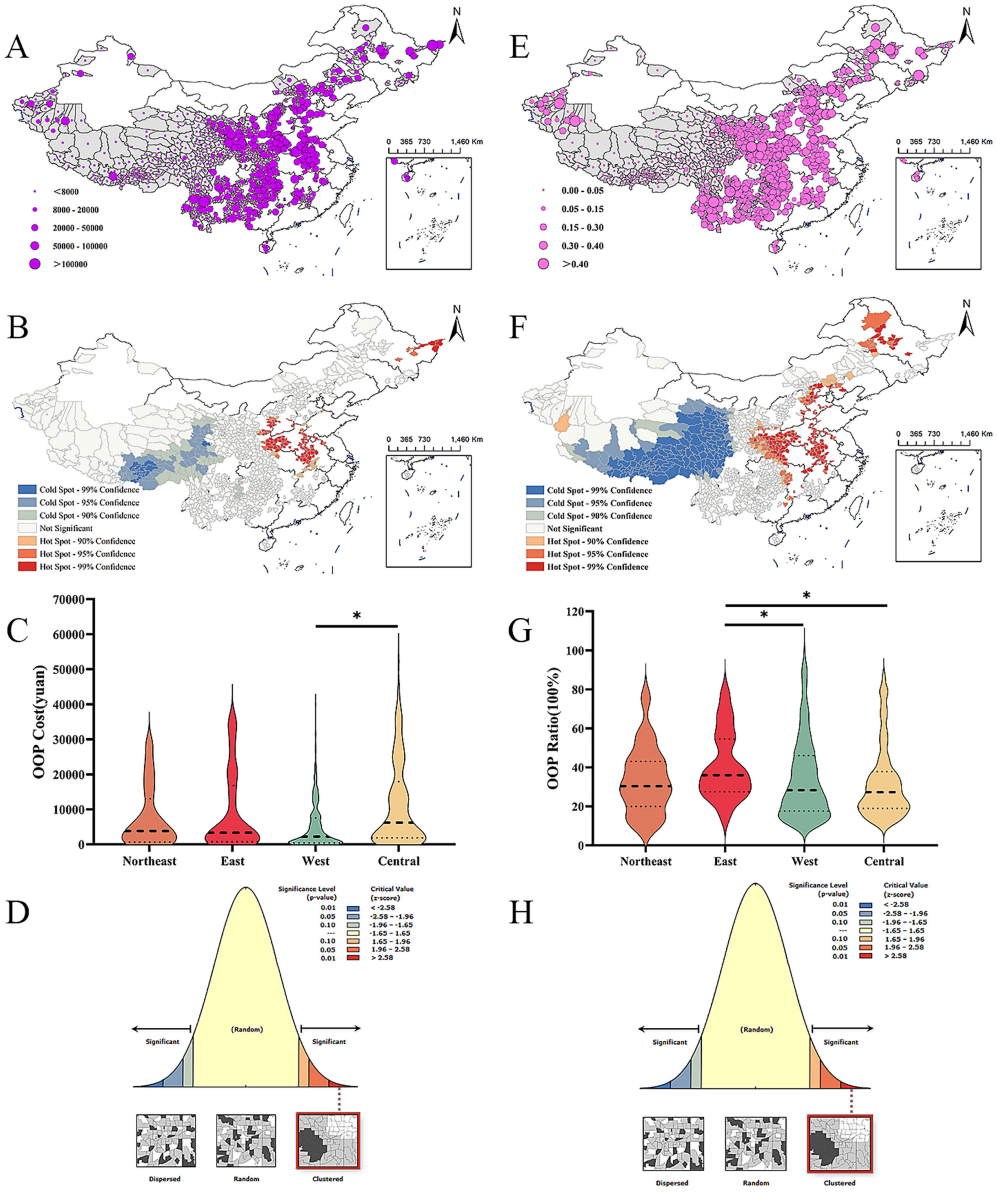
Spatiotemporal distribution and spatial autocorrelation of leukemia out-of-pocket (OOP) costs and OOP ratios (2019–2024). **(A–D)** OOP costs statistical analyses. **(A)** Spatiotemporal distribution. High OOP cost counties (≥50,000 RMB/year) were consistently concentrated in Central China and parts of East China; low OOP cost counties (<20,000 RMB/year) dominated in West China. **(B)** Getis-Ord Gi* hotspot analysis. Hotspots of high OOP costs were concentrated in Central China; no significant hotspots were observed in West China. **(C)** Regional comparison of median OOP costs. Central China had the highest median OOP cost, followed by East and Northeast China; West China had the lowest. Differences between Central and West China were statistically significant, while East and Northeast China showed no significant difference. **(D)** Global Moran’s I for OOP costs. Significant spatial clustering was observed, indicating that counties with similar OOP costs tended to be geographically adjacent. **(E–H)** OOP ratios statistical analyses. **(E)** Spatiotemporal distribution. High OOP ratio counties (≥30%) were concentrated in East, Northeast, and Central China; low OOP ratio counties (<15%) were primarily in West China. **(F)** Getis-Ord Gi* hotspot analysis. Hotspots of high OOP ratios were distributed in East and Central China; coldspots dominated West China. **(G)** Regional comparison of median OOP ratios. East China had the highest median OOP ratio (39.7%), approaching the WHO catastrophic health expenditure threshold (40%); this was significantly higher than West China (22.3%, *p* < 0.01) but not different from Northeast China (37.1%, *p* > 0.05). **(H)** Global Moran’s I for OOP ratios. Stronger spatial clustering was observed compared to OOP costs (Moran’s I = 0.189, *p* < 0.001), reflecting pronounced regional disparities in financial toxicity. OOP costs = sum of direct medical expenditures for leukemia treatment; OOP ratios = OOP costs/total household health expenditure × 100%. All spatial analyses used a fixed bandwidth = 200 km and Queen neighborhood criterion; statistical significance was determined by Monte Carlo permutation tests (*p* < 0.01).

Counties with high OOP costs were mainly concentrated in the Central region (Figure 6A). In contrast, counties with low OOP costs were primarily located in the Western region (Figure 6A). Per capita OOP costs in the Central region were higher than those in the Western region. However, no statistically significant difference was observed when comparing the Central region with the Eastern and Northeastern regions (Figure 6C). The results of the global spatial autocorrelation analysis [Moran I = 0.104329, *p* < 0.001] indicated a significant aggregation pattern (Figure 6D). Furthermore, the optimized hotspot analysis revealed that hotspot regions were predominantly situated in the Central, Northeast, and East regions, whereas coldspot regions were primarily found in certain areas of the western part (Figure 6B).

Regions with elevated OOP ratios were predominantly located in the East, Northeast, and Central regions, while areas with lower OOP rates were primarily found in the West (Figure 6E). Eastern China exhibited the highest financial burden with a median Out-of-Pocket (OOP) ratio of 39.7%, critically approaching the WHO catastrophic threshold (7). Regional comparison revealed significantly higher OOP ratios in the East than West/Central (*p* < 0.001), while no statistical difference existed between East and Northeast as quantified in Figure 6G. The global spatial autocorrelation analysis indicated a significant global autocorrelation [Moran I = 0.188918, *p* < 0.001] in the OOP ratio, revealing a notable clustering pattern and positive spatial autocorrelation (Figure 6H). Furthermore, the optimized hotspot analysis identified hotspot regions primarily in the Central and Northeastern regions, while coldspot regions were predominantly located in the West (Figure 6F).

## 4 Discussion

This study represents the first epidemiological spatio-temporal analysis of leukemia among a population of 832 poverty-eradication counties across four major economic regions of China from 2019 to 2024. It holds significant importance for enhancing the targeting of leukemia prevention and control efforts, as well as for improving the effectiveness of preventing a resurgence of poverty attributable to leukemia.

The incidence of leukemia is significantly higher among the impoverished population in the eastern and northeastern regions compared to the western regions, particularly during the period from 2019 to 2021. This disparity may be attributed to various factors, including environmental conditions, lifestyle choices, and inadequate access to healthcare. Industrial pollution is a significant contributor to the elevated incidence of leukemia in the eastern region (2, 5), where individuals are more frequently exposed to harmful substances such as benzene. This exposure damages bone marrow and leads to chromosomal abnormalities, thereby increasing the likelihood of individuals developing leukemia (8–10). Secondly, the accelerated urbanization in the eastern region has resulted in significant lifestyle changes, with dietary patterns marked by high intake of fats, sugars, and salts recognized as a known risk factor for various types of leukemia, particularly chronic lymphocytic leukemia and granulocytic leukemia (11–13). Additionally, inadequate healthcare infrastructure, limited access to healthcare services (14), and low awareness of leukemia symptoms (15) may contribute to delayed diagnosis and increased morbidity. To address this issue, several measures have been implemented in the eastern region, including enhancing public health awareness and knowledge related to leukemia, promoting healthy lifestyles, strengthening environmental protection to mitigate chemical and environmental pollution (16, 17), screening and monitoring at-risk groups to identify and address potential issues, and establishing health policies and interventions tailored to the specific needs of the population in eastern China. The specific needs of the population in eastern China are addressed in reference (2). Improved access to healthcare has enabled more individuals to receive timely diagnoses and treatments (18). Furthermore, advancements in medical testing and diagnostic technologies have greatly enhanced the capacity to identify leukemia (5). As the economy progresses, the integration of advanced medical technologies and treatment options, such as targeted therapies and hematopoietic stem cell transplants, has markedly improved patient survival rates and prognoses (2).

Crucially, this observed hotspot expansion in western China post-2022, as identified by our Getis-Ord Gi analysis (Figure 5), stands in apparent contrast to the significant declines in national leukemia incidence and mortality reported herein (Table 1). This phenomenon presents the paradox of ‘high resource allocation leading to an apparent high disease burden’ at the local level and warrants specific consideration in relation to Health Poverty Alleviation (HPA) policy impacts. The post-2022 expansion of hotspots (Figure 5) reflects the progressive rollout of HPA interventions reaching deeper into Western regions, unmasking the pre-existing, but previously undiagnosed, burden of leukemia. This increased detection directly results from higher resource investment aimed at improving health equity. Thus, the seemingly paradoxical emergence of ‘high burden’ (hotspots) in the West following ‘high resource’ (HPA) investment is not indicative of a failure of the policies or an actual worsening of leukemia burden, but rather a testament to their success in improving case ascertainment and bringing previously hidden cases into the healthcare system. This phenomenon aligns with the identified peak in 2020 being attributable to uncovered backlogged cases (discussed earlier), but demonstrates its spatio-temporally evolving nature, shifting focus to disadvantaged western regions as HPA interventions deepened after 2020. Before 2022, the basic medical facilities in western China were relatively weak (19), with a lower number of health technicians per thousand population compared to the eastern region (20). This situation severely impacted the quality and accessibility of medical services. Consequently, leukemia’s were more likely to develop (21) and were often diagnosed at later stages, leading to higher morbidity and mortality rates (22) between 2019 and 2021 (23). However, following economic development and the poverty alleviation of impoverished counties (24), significant improvements have been made. With advancements in diagnostic capabilities and timeliness (25), the detection rate of leukemia has steadily increased in poverty-stricken regions of Western China (26). Getis-Ord Gi* analysis revealed significant hotspot expansion in western China post-2022 (Figure 5), contrasting with the prior northeast-central clustering pattern.

The distribution of the economic burden of leukemia is markedly uneven, with the eastern and central regions facing a greater economic burden than the poorer western regions, despite having better access to healthcare resources. With the OOP ratio median (39.7%) nearing the WHO catastrophic threshold (40%) (27), eastern China exhibits critical vulnerability to financial toxicity despite higher economic development (Figure 6). This suggests systemic inefficiencies in public coverage for leukemia care, demanding urgent formulary expansion to novel therapies. This high economic burden in the eastern and central regions may stem from a cycle characterized by “high healthcare resources - high treatment demand.” The advanced medical facilities and specialists available in these regions provide better treatment options and outcomes for leukemia patients, who often have elevated expectations regarding their treatment (28). However, the depth of health insurance coverage is frequently inadequate to fully cover the costs associated with leukemia-related treatments. Consequently, the involvement of insurance companies can lead to catastrophic medical expenditures, which significantly impact impoverished families (29).

Quality healthcare resources tend to attract more complex cases, such as those requiring hematopoietic stem cell transplantation. Conversely, inadequate coverage in health insurance catalogs compels patients to pay OOP for innovative drugs (30). This significant financial burden can heighten stress and anxiety for both patients and their families, complicating the disease management process (5, 31) and potentially leading families to abandon treatment, which can have severe consequences (32). In the western region, characterized by poorer economic conditions and medical standards (19, 33, 34), patients generally have lower treatment expectations and rely more heavily on local medical resources. This reliance often results in poorer treatment outcomes and higher mortality rates (35, 36) despite the relatively lower financial burden (37).

To address the significant financial burden in the east-central region, a pilot leukemia-specific healthcare voucher could be implemented to cover the OOP expenses associated with new targeted therapies. Additionally, expanding the scope of healthcare insurance and promoting the integration of commercial insurance with basic healthcare insurance would provide health-related financial risk protection for the impoverished, thereby reducing the likelihood of further poverty due to catastrophic healthcare expenditures (38, 39). Furthermore, the reimbursement rate for leukemia treatments should be increased, and a cross-provincial medical collaboration network should be established to lower the costs associated with inter-regional patient mobility. To tackle the issue of inadequate healthcare resources in the western region, the government should prioritize investments in enhancing healthcare facilities and services, elevating the level of medical assistance in impoverished counties, and ensuring that all individuals have access to basic medical insurance, major disease insurance, and medical assistance (40, 41). Lastly, efforts to improve public health literacy should be intensified to enhance overall health outcomes and decrease the incidence of disease-related poverty (42, 43).

## 5 Innovations

Transcending the provincial-scale spatial analyses dominating leukemia burden research and unidimensional economic evaluations, this study introduces three integrated innovations: First, a granular spatiotemporal framework analyzes county-level dynamics (*n* = 832 counties) through terrain-adjusted spatial autocorrelation (Global Moran’s I; Getis-Ord Gi*) coupled with Joinpoint regression (BIC-optimized inflection detection). Second, we establish a dual-burden quantification system, capturing both absolute OOP costs and relative financial toxicity (OOP ratios), revealing significant inter-regional disparities. Crucially, we reframe 2022–2024 high-burden clusters not as policy failures but as evidence of diagnostic catch-up success, where spatial case ascertainment bias serves as an equity-sensitive metric for healthcare modernization impact.

## 6 Limitations

Although this study systematically assessed the spatial distribution characteristics of leukemia incidence and its economic burden, the following limitations remain: 1. Data coverage bias: the data from the poverty alleviation platform may miss unregistered poor cases, especially in remote areas with poor transportation, which may lead to underestimation of incidence and distortion of spatial patterns. Follow-up studies need to validate patient coverage by combining data from multiple sources 2. Missing indirect costs: The economic analysis did not include indirect costs such as missed work and companionship, which may weaken the comprehensive assessment of the poverty-causing effects of the disease 3. Environmental and socioeconomic covariates were not fully captured and could influence spatial disparities. 4. Incomplete longitudinal socioeconomic indicators (832 counties, 2019–2024), absent age-stratified incidence matrices, and missing non-demographic covariates precluded three advanced analyses: SII/RII quantification of contextual disparities, APC modeling of cohort effects, and decomposition of population/non-population drivers. These gaps restrict cross-validation with studies using such causal inference techniques. Future work requires high-granularity socioeconomic panels, demographically disaggregated registries, and geospatial covariate libraries.

## Data Availability

The data analyzed in this study is subject to the following licenses/restrictions: you can get access to the data by requesting it from corresponding author. Requests to access these datasets should be directed to zllrmit@aliyun.com; qydelwp@foxmail.com.
